# Bioactivities and Health Benefits of Mushrooms Mainly from China

**DOI:** 10.3390/molecules21070938

**Published:** 2016-07-20

**Authors:** Jiao-Jiao Zhang, Ya Li, Tong Zhou, Dong-Ping Xu, Pei Zhang, Sha Li, Hua-Bin Li

**Affiliations:** 1 Guangdong Provincial Key Laboratory of Food, Nutrition and Health, School of Public Health, Sun Yat-Sen University, Guangzhou 510080, China; zhangjj46@mail2.sysu.edu.cn (J.-J.Z.); liya28@mail2.sysu.edu.cn (Y.L.); zhout43@mail2.sysu.edu.cn (T.Z.); xudp@mail2.sysu.edu.cn (D.-P.X.); zhangp@mail2.sysu.edu.cn (P.Z.); 2 School of Chinese Medicine, The University of Hong Kong, Hong Kong 999077, China; u3003781@connect.hku.hk; 3 South China Sea Bioresource Exploitation and Utilization Collaborative Innovation Center, Sun Yat-Sen University, Guangzhou 510006, China

**Keywords:** mushroom, bioactivity, antioxidant, anticancer, anti-inflammation

## Abstract

Many mushrooms have been used as foods and medicines for a long time. Mushrooms contain polyphenols, polysaccharides, vitamins and minerals. Studies show that mushrooms possess various bioactivities, such as antioxidant, anti-inflammatory, anticancer, immunomodulatory, antimicrobial, hepatoprotective, and antidiabetic properties, therefore, mushrooms have attracted increasing attention in recent years, and could be developed into functional food or medicines for prevention and treatment of several chronic diseases, such as cancer, cardiovascular diseases, diabetes mellitus and neurodegenerative diseases. The present review summarizes the bioactivities and health benefits of mushrooms, and could be useful for full utilization of mushrooms.

## 1. Introduction

Edible mushrooms are regarded as healthy food and nutrient sources because of their many beneficial components, including carbohydrates, dietary fiber, protein, vitamins and minerals, and low levels of calories, fat and toxic metals [1,2]. Moreover, human beings have used mushrooms as medicines for 5000 years or more [3]. It has been demonstrated that numerous mushrooms have remarkable bioactivities, including antioxidant, antitumor, antiviral, anti-inflammatory, and immunoregulatory effects [4]. Recently, medicinal mushrooms have attracted more and more attention as potential natural agents for the prevention and treatment of many diseases, such as cancer, cardiovascular diseases, diabetes mellitus and neurodegenerative diseases. This review summarizes current knowledge of mushroom bioactivities, including their antioxidant, anticancer, immunomodulatory, anti-inflammatory, and antimicrobial activities, which could be helpful for the full utilization of mushrooms.

## 2. Antioxidant Activity

Excess production of free radicals can cause damage to DNA, lipids and proteins, which in turn can result in several chronic diseases, such as cardiovascular diseases, cancer and neurodegenerative diseases. Various natural products, such as vegetables, fruits, edible flowers, cereal grains and medicinal plants, contain rich natural antioxidants [5,6,7,8,9], which can capture free radicals and be used to prevent some diseases caused by oxidative stress. Among the notable medicinal properties of mushrooms, the antioxidant activity, including inhibition of lipid peroxidation, reduction of human low-density lipoproteins, scavenging of free radicals, etc., has been extensively studied.

Polysaccharides in mushrooms are generally considered to be the main contributors to the antioxidant activity. A water-soluble polysaccharide isolated from *Inonotus obliquus* was studied and the results showed that the polysaccharide was an acid protein-bound polysaccharide, with a molecular weight of 17 kDa and its contents of neutral sugars, protein and uronic acids were 42.5%, 18.5% and 6.1%, respectively. The results indicated that the polysaccharide had inhibitory activity evidenced by concentration-dependent quenching of DPPH and hydroxyl radicals. Additionally, the polysaccharide inhibited the formation of thiobarbituric acid-reactive substances in Fe^2+^/ascorbate-induced lipid peroxidation in rat liver tissue. All this demonstrated that *Inonotus obliquus* had antioxidant effects [10]. In another study, polysaccharides from Jisongrong mushroom also possessed strong antioxidant and antitumor activity. The Jisongrong polysaccharides were orally administrated to rats for 2 months. Compared to control rats, levels of lipid peroxidation products and activities of antioxidant enzymes in the blood were significantly decreased and enhanced, respectively. Furthermore, Jisongrong polysaccharides markedly inhibited cancer cell proliferation [11]. In addition, the key chemical constituents and antioxidant activities of water-soluble polysaccharide fractions isolated from three edible mushrooms were investigated. The results indicated that the antioxidant activities of all polysaccharide fractions were significantly correlated with the total phenolic and protein contents, but not with the carbohydrate contents. Purified polysaccharides free of phenolic compounds and protein had no significant activities. Thus, the phenolic and protein components instead of carbohydrates were mainly responsible for the antioxidant activities of mushroom polysaccharides [12]. In another study, crude polysaccharides isolated from four common edible mushrooms including *Agaricus bisporus*, *Auricularia auricula*, *Flammulina velutipes* and *Lentinus edodes* were studied. The crude polysaccharides of *Agaricus bisporus* were the best natural antioxidant [13]. Water extracts from five mushrooms were also analyzed. The results showed that the antioxidant order was *Inonotus obliquus* > *Ganoderma lucidum* > *Lentinus edodes* > *Tremella fuciformis* > *Auricularia auricula*. Ethanol extracts from *Inonotus obliquus* exhibited the highest antioxidant activity among the five mushrooms tested [14], making *Inonotus obliquus* a novel antioxidant candidate.

The antioxidant activities of selenium- and zinc-enriched mushrooms (SZMs) have been widely evaluated. The antioxidant power of SZMs was examined by measuring the activities of antioxidant enzymes and the levels of lipid peroxide products in mice. The study showed that treatment with SZMs meaningfully improved the activities of glutathione peroxidase and superoxide dismutase, and decreased the levels of malondialdehyde and lipofuscin. Thus, SZMs might be effective for increasing antioxidant capacity [15]. Furthermore, *Ganoderma lucidum* could biotransform inorganic selenium into organic selenium, which was stored preferentially in its water-soluble protein compounds. In a separate study, the relationship between the antioxidant activity of proteins in *Ganoderma lucidum* and its Se contents was investigated. The protein with higher Se showed approximately three times stronger activity scavenging superoxide and hydroxyl radicals as compared to the water-soluble protein extracts. It was demonstrated that the increasing antioxidant property of this protein in *Ganoderma lucidum* depended quantitatively on its Se contents [16].

Several types of mushrooms collected from southwest China have been studied. These mushrooms (*Clitocybe maxima*, *Catathelasma ventricosum*, *Stropharia rugosoannulata*, *Craterellus cornucopioides* and *Laccaria amethystea*) contained beneficial bioactive compounds such as phenols, ergosterol, tocopherol, ascorbic acid, unsaturated fatty acids and essential amino acids, which could be used as antihyperglycemic and antioxidant ingredients [17]. Particularly, *Catathelasma ventricosum* and *Laccaria amethystea* were effective in the protection against hyperglycemia and oxidative stress [17]. In addition to the above mushrooms, some popular medicinal mushrooms in Asia have been studied extensively. *Cordyceps militaris* is one of the most valuable medicinal mushrooms and nutraceuticals in China. A study assayed the antioxidant activities of the methanol extracts from the fruiting bodies of *Cordyceps militaris*. The research indicated that the antioxidant potential of the extracts was significant in the four tested systems in vitro, including total antioxidant capacity, scavenging ability on DPPH radicals, reducing power, and chelating ability on ferrous ions [18]. Besides, Wang et al. studied the effects of cordymin (a peptide purified from *Cordyceps militaris*) on prevention of focal cerebral ischemic/reperfusion injury in rats [19]. Administration (oral) of cordymin significantly boosted the defense mechanism against cerebral ischemia by increasing antioxidant activity. It was demonstrated that cordymin in *Cordyceps militaris* had potential antioxidant activity. Shiitake (*Lentinula edodes*), which is famous for its high nutritional value and medicinal properties, is the second most cultivated mushroom. The chemical constituents and antioxidant power of shiitake are significantly affected by the drying method. The study indicated that hot air drying at 50 °C resulted in high total phenolic content and antioxidant activity [20]. *Ganoderma sinensis* is an endemic mushroom in China and has been used as medicine or food for centuries. *Ganoderma sinensis* was available in form of log-cultivated and sawdust-cultivated fruit bodies, solid-fermented products and liquid-fermentation mycelia. Methanolic and hot water extracts of these four forms were prepared and then their antioxidant properties were investigated. It was observed that both extracts from the four forms of *Ganoderma sinensis* exhibited high antioxidant activities of 69.69%–99% at 20 mg extract/mL and low EC_50_ values of 0.95–10.00 mg extract/mL. Moreover, both extracts could scavenge hydroxyl radicals [21]. In another study, blood samples of seven healthy volunteers were collected, and total antioxidant activity of plasma was evaluated before and after each treatment of *Ganoderma sinensis*. The research indicated that intake of *Ganoderma sinensis* significantly increased the antioxidant activity of plasma [22]. In addition, medicinal mushroom *Inonotus obliquus* is a traditional and widely used multi-functional fungus, and has shown strong antioxidant activity [23]. Numerous studies have thus been carried out on the antioxidant effects of mushrooms. The antioxidants activities of some mushrooms not mentioned above are summarized in Table 1.

## 3. Anti-Inflammatory Activity

Medicinal mushrooms have been indispensable components of traditional Chinese herbal medicines for thousands of years [32]. Anti-inflammatory activity is an essential property of medicinal mushrooms to promote healthy effects.

*Cordyceps sinensis* has been used as a functional food and herb for a long time. The anti-inflammatory effect of *Cordyceps sinensis* components has been investigated, especially cordymin, a peptide purified from *Cordyceps sinensis*. There is a study about the effects of cordymin on prevention of focal cerebral ischemic/reperfusion injury. In the experiment, the right middle cerebral artery occlusion model was used. Rats were treated with cordymin orally. This finding showed that cordymin had a neuroprotective effect in the ischemic brain, which was attributed to the inhibition of inflammation and enhancement of antioxidant activity related to lesion pathogenesis [19]. In another study, the effects of cordymin on cytokine levels and total antioxidant activity were analyzed. The antinociceptive effects of cordymin in vivo and in vitro were also examined. The levels of tumor necrosis factor-alpha (TNF-α), interleukin 1 beta and total antioxidant status were decreased after cordymin treatment. Cordymin also inhibited the acetic acid-induced abdominal constrictions in mice in a dose-dependent manner. Besides, cordymin exhibited strong activities against neurolysin (IC_50_ = 0.1 µM) in neurolysin inhibition assay. It was concluded that cordymin was a potent anti-inflammatory medicine [33].

*Inonotus obliquus* mushroom is regarded as a precious traditional Chinese herb as well. The anti-inflammatory and anticancer constituents in *Inonotus obliquus* were identified by bioassay-guided preparative isolation. The petroleum ether and ethyl acetate fractions were observed to have meaningful inhibition effects on nitric oxide production and NF-κB luciferase activity in RAW 264.7 macrophage cells. Three constituents isolated from these two fractions—ergosterol, ergosterol peroxide and trametenolic acid—exhibited anti-inflammatory activities [34].

Some other components derived from medicinal mushrooms also show strong anti-inflammatory activities. Hispidin, a polyphenol component mainly derived from medicinal *Phellinus* mushroom species, has been proved to possess distinct biological properties [35]. The research showed that hispidin inhibited transcriptional activity of NF-κB in a dose-dependent manner. It was also observed that hispidin attenuated LPS-induced NF-κB nuclear translocation and associated inhibitor of IêB-á degradation. Moreover, hispidin reduced iNOS protein expression and the generation of reactive oxygen species (ROS) in the LPS-induced cells, but phosphorylation of mitogen-activated protein kinases was not affected. These findings indicated that hispidin presented anti-inflammatory activity by suppressing ROS mediated NF-κB pathway [35]. Besides, the anti-inflammatory effect of the polysaccharides isolated from golden needle mushroom was investigated in burned rats. The results showed that the polysaccharides possessed strong anti-inflammatory effects [36]. Moreover, a new fungal secondary metabolite, agaricoglycerides from royal sun medicinal mushroom, was investigated. It was demonstrated that hepatic glycemic metabolism dysfunction, inflammation, and oxidative stress in mice were alleviated after administration of agaricoglycerides. These data showed that the agaricoglycerides in royal sun mushroom had the effects of decreasing the levels of inflammatory cytokines [37].

## 4. Immunomodulatory Activity

Immunomodulatory activity is considered as a critical factor of protecting humans against many diseases. Many mushrooms such as *Lentinus edodes*, *Schizophyllum commune*, *Grifola frondosa*, and *Ganoderma lucidum* are important natural sources of immunomodulatory agents [38].

The ability of mushrooms to modulate immune functions is mostly attributable to their bioactive compounds, including polysaccharides, proteins, proteoglycans and triterpenoids [38]. Mushroom polysaccharides were extensively studied for their immunomodulatory activities. There was a study about the potential effects of polysaccharides from medicinal mushroom *Amauroderma rude* on immune regulation. Results showed that crude extract of *Amauroderma rude* increased the activities of spleen lymphocytes, macrophages, and natural killer cells in vitro, and increased macrophage metabolism, lymphocyte proliferation, and antibody production in vivo. In addition, the active compound in the crude extract was purified and identified as polysaccharide F212 [39]. Polysaccharides from the mushroom *Dictyophora indusiata* were also studied. The research showed that the polysaccharides could promote macrophage multiplication. In other words, the polysaccharides significantly affected immune functions by prompting the production of nitric oxide and cytokines, such as tumor necrosis factor-alpha (TNF-α), interleukin-1, -6, and -12 [40]. Proteins from the mushrooms were also studied, because bioactive proteins were regarded as an important group of functional agents in medicinal mushrooms. A new immunomodulatory protein from *Trametes versicolor*, named TVC, was studied. TVC could enhance the proliferation of splenocytes, while it had no stimulatory effects on CD4^+^ and CD8^+^ T cells in biological activity assays. Furthermore, TVC significantly increased the proliferation of human peripheral blood lymphocytes in a dose-dependent manner and improved the production of both nitric oxide and TNF-α by lipopolysaccharide-induced murine macrophages [41]. Besides, FIP-fve, isolated from the mushroom *Flammulina velutipes*, is a bioactive protein which was demonstrated to possess several kinds of biological activities, including anti-allergy, anti-tumor and immunomodulation properties [42].

Many studies have been carried out on immune function effects of *Ganoderma lucidum*. In a study, a novel polysaccharide (TB3-2-2) was successfully isolated and purified from *Ganoderma lucidum*. TB3-2-2 significantly increased the proliferation of mouse spleen lymphocytes and the expression levels of interleukin-6. It was demonstrated that the polysaccharide in *Ganoderma lucidum* mushroom might have the immune regulation potential [43]. LZ-B-1, a water-soluble peptidoglycan was also purified from the fruiting bodies of *Ganoderma lucidum*. In vitro experiments showed that LZ-B-1 promoted proliferation of mouse spleen lymphocytes (MSLs) with an optimum concentration of 200 µg/mL. Generally, the higher the mouse spleen cell proliferation rate, the stronger the immunomodulatory activity, therefore the peptidoglycan LZ-B-1 had immunomodulatory effect [44]. There was also a study about the immunomodulatory effects of a diet supplemented with *Ganoderma lucidum* mycelium. Over 14 weeks, mice from the test group were fed with two concentrations of *Ganoderma lucidum* mycelium, at 85% or 50%, labeled G85 and G50 diets. In contrast, mice from the control group received a regular diet. *Ganoderma lucidum*-supplemented diets significantly altered the immune system of the mice (*p* < 0.05), and the G50 diets were more effective than the G85 diets. These results indicated that *Ganoderma lucidum* had immunomodulatory activity [45].

## 5. Anticancer Activity

Cancer is a leading cause of death around the world. Recently, the need for more effective and safe treatments for chemoprevention of human cancer has increased. Some natural products, such as fruits and medicinal plants, have shown antiproliferative activities [46,47,48]. Numerous studies have demonstrated that mushrooms show significant inhibitory activity against breast cancer, hepatocellular carcinoma, uterine cervix cancer, pancreatic cancer, gastric cancer and acute leukemia. In addition, antitumor compounds have been identified in various mushrooms species [49].

Because breast cancer is the major cause of death among women, there are many studies about mushrooms’ activities against breast cancer. Three triterpenoids (2,3,6,23-tetrahydroxy-urs-12-en-28-oic acid, 2,3,23-trihydroxy-urs-12-en-28-oic acid and lupeol) were isolated from the mushroom *Pleurotus* *eryngii*. The study indicated that the three triterpenes possessed significant inhibitory activity against breast cancer MCF-7 cell lines in vitro, with the greatest activity exhibited by 2,3,6,23-tetrahydroxy-urs-12-en-28-oic acid [50]. Besides, a carboxymethylated P-glucan (CMPTR) from the mushroom sclerotia of *Pleurotus tuberregium* was studied using human breast carcinoma MCF-7 cells in vitro. CMPTR induced anti-proliferative activity dose-dependently, with an IC_50_ of 204 µg/mL, and inhibited the cell proliferation of MCF-7 by arresting the G_1_ phase of its cell cycle. In addition, the CMPTR-treated MCF-7 cancer cells were associated with decreased expression of anti-apoptotic Bcl-2 protein and increased expression of Bax/Bcl-2 ratio. The research indicated that carboxymethylated P-glucan could inhibit the proliferation of breast cancer MCF-7 by cell-cycle arrest and apoptosis induction [51]. In another study, both ergosterol peroxide and 9,11-dehydroergosterol peroxide isolated from *Ganoderma lucidum* also exhibited inhibitory effects on human breast adenocarcinoma MCF-7 cells by inducing cell apoptosis [52]. Furthermore, evidences from a meta-analysis of observational studies demonstrated that mushroom intake might be inversely associated with risk of breast cancer [53].

Mushrooms have become a focus of interest in the treatment of hepatocellular carcinoma (HCC). A study showed that polysaccharides purified from the mushroom *Trametes robiniophila* (huaier) not only meaningfully inhibited the proliferation of SMMC-7721 cells in vitro, but also suppressed the HCC tumor growth and metastatic nodules to the lung in SMMC-7221-bearing mice by oral administration [54]. Concomitantly, immune histochemistry analysis of tumor tissues indicated that polysaccharides administration at three doses significantly inhibited the cancer cell proliferation in vivo. Taken together, this study showed that these polysaccharides might be a promising chemopreventive agent for the tumorigenesis and metastasis of HCC [54]. Besides, recent studies have demonstrated that the *Coriolus versicolor* (turkey tail) polysaccharides (CVPs) could inhibit the proliferation of cancer cells in vitro and in vivo, and different purity levels of CVPs had different effects on various cancer cells. The cytotoxic activity of the CVPs was investigated in vitro on a human hepatoma cancer (QGY) cell line by using the MTT assay. The cell cycle and cell apoptosis of QGY cells were examined by flow cytometry. The results showed that the CVPs inhibited the proliferation of human hepatoma cancer in low concentration (<20 mg/L) with an IC_50_ of 4.25 mg/L, and a significant decrease in the expression of the cell cycle-related genes (*p*53, Bc1-2, and Fas) in these cells was observed. Therefore, the CVPs isolated from turkey tail mushroom could be a potential candidate to ameliorate toxic effects in cancer therapy [55]. In addition, some experiments have demonstrated that suillin from the mushroom *Suillus placidus* might be an effective agent to treat liver cancer by inducing apoptosis in human hepatoma HepG2 cells [56].

Moreover, specific popular mushrooms had significant effects on other cancers. *Cordyceps sinensis* is an extensively used medicinal mushroom in China. The effect of fermented *Cordyceps sinensis*, rich in selenium (Se-CS), on uterine cervical cancer in mice was studied. The methylcholanthrene (MCA)-induced group showed 85.7% tumor incidence, and the animals showed 40% tumor incidence (*p* < 0.05) after administrating the mice with Se-CS. This finding indicated that Se-CS could be a potential treatment for uterine cervical cancer [57]. Another valuable medicinal mushroom *Ganoderma lucidum* was studied using the tumorigenic transformable human urothelial cell (HUC-PC) model. The data showed that *Ganoderma lucidum* could inhibit the viability and the growth of HUC-PC in vitro by induction of apoptosis and suppression of telomerase activity. Therefore, *Ganoderma lucidum* was identified as a potential source of chemopreventive agents for bladder cancer [58]. Mushrooms could also be antitumor agents of acute leukemia and gastric cancer [59,60].

The potential for inhibiting tumor growth of thirteen mushrooms was screened, and the study showed that the water extract of *Amauroderma rude* exerted the highest anticancer activity, decreasing tumor weight by about 50% and 70% as well as tumor volume 40% and 75% at low and high dosages, respectively [39]. In addition the compound ergosterol was purified from *Amauroderma rude*. In in vivo research, the survival times of normal mice injected with the aggressive murine cancer cell line B16 were prolonged by treatment with ergosterol, indicating that ergosterol might be the leading anticancer substance in *Amauroderma rude* [61]. *Cordyceps taii*, an entomogenous fungus native to south China, is a common medicine with a variety of pharmacological activities, including anticancer effects. The cytotoxic activities of chloroform extract of *Cordyceps taii* (CFCT) against human lung cancer (A549) and gastric cancer (SGC-7901) cells in vitro were examined by a sulforhodamine B (SRB) assay. Kunming mice- bearing sarcoma 180 and C57BL/6 mice bearing melanoma B16F10 were employed to study its antitumor and anti-metastatic activities. The results showed that CFCT exhibited dose-and time-dependent cytotoxicity against A549 and SGC-7901 cells, and could significantly inhibit the tumor growth in vivo and prolong the survival time in two different models compared with the model group [62]. The mushroom *Inonotus obliquus* has been traditionally used as a folk remedy for the treatment of cancers [63]. It was found that petroleum ether and ethyl acetate fractions of *Inonotus obliquus* had cytotoxicity against human prostate carcinoma cell PC3 and breast carcinoma cells MDA-MB-231 [34]. The anticancer mechanism of *Inonotus obliquus* was investigated. Several components isolated from *Inonotus obliquus* arrested cancer cells in the G_0_/G_1_ phase and then induced cell apoptosis or differentiation, while several components directly participated in the cell apoptosis pathway. In addition, polysaccharides from *Inonotus obliquus* could indirectly be involved in anticancer processes mainly via stimulating the immune system. Besides, the antioxidants of *Inonotus obliquus* extracts could inhibit generation of cancer cells [64]. In Taiwan, *Antrodia cinnamomea* is a valuable medicinal mushroom popularly used in adjuvant cancer therapies, and its major bioactive compounds were ergostanes, lanostanes and triterpenoids [65].

Investigators have found many novel substances derived from valuable mushrooms, and their anticancer effects were studied. A new hemagglutinin was isolated from mushroom *Boletus speciosus*. In in vitro research, the hemagglutinin showed antiproliferative activity towards hepatoma Hep G2 cells and mouse lymphocytic leukemia cells (L1210) [66]. Besides, a compound, named jiangxienone, was extracted from the traditional Chinese medicinal mushroom *Cordyceps jiangxiensis*. This study indicated that jiangxienone exhibited potent cytotoxic effects against human gastric adenocarcinoma SGC-7901 cells and human lung carcinoma A549 cells [67]. Finally, the anticancer activities of some mushrooms not mentioned above are given in Table 2.

## 6. Antimicrobial Effects

Over the past century, numerous synthetic antimicrobial agents have been discovered and developed, but drug resistance and toxicity are still the major hindrances to gaining achieving therapeutic effects [75]. Therefore, it is necessary to seek safe and useful agents for infectious diseases. Herbal medicine ingredients, such as mushrooms, are considered a reliable resource in this context. Various mushrooms have demonstrated potential antibacterial, antifungal and antiviral activities.

In one study, the antimicrobial effects of crude extract of the culinary-medicinal mushroom *Auricularia auricular-judae* were tested against some bacteria and fungi. It was observed that the extract showed antibacterial effects towards *Escherichia coli* and *Staphylococcus aureus*, but no effects on other species. The diameters of antimicrobial inhibition zones for the two species were 5.55 ± 0.182 and 9.84 ± 0.076 mm, respectively. This research indicated that crude extract of *Auricularia auricular-judae* had a great potential as an antimicrobial [76]. In another study, a water-soluble polysaccharide named PL, isolated and purified from a spent mushroom substrate, was studied for the antibacterial activity against *Escherichia coli*, *Staphylococcus aureus* and *Sarcina lutea*, and the minimal inhibitory concentrations (MICs) were established. The polysaccharide, which contained two fractions (PL1 and PL2), showed the strongest antibacterial activity against *Escherichia coli*, while it showed the weakest activity against *Sarcina lutea*, with MICs of 12.5, 25 and 100 µg/mL for *Escherichia coli*, *Staphylococcus aureus* and *Sarcina lutea*, respectively [77]. In addition, a novel antibacterial protein, with a molecular mass of 44 kDa, has been isolated from dried fruiting bodies of the wild mushroom *Clitocybe sinopica*. It has been shown that the protein was composed of two subunits, each with a molecular mass of 22 kDa, by sodium dodecyl sulfate/polyacrylamide gel electrophoresis. The results indicated that the protein possessed potent antibacterial activities against *Agrobacterium rhizogenes*, *A. tumefaciens*, *A. vitis*, *Xanthomonas oryzae* and *X. malvacearum* and the minimum inhibitory concentrations were mostly below 0.6 µM, while the protein showed no antibacterial effects against *Pseudomonas batatae*, *Erwinia herbicola*, *Escherichia coli*, and *Staphylococcus aureus*, and no antifungal activities against *Setosphaeria turcica*, *Fusarium oxysporum*, *Verticillium dahliae*, *Bipolaris maydis*, or *B. sativum* [78].

*Ganoderma* *lucidum* was used to treat chronic infectious diseases, such as chronic hepatitis and bronchitis in Asia [79]. In vitro and in vivo preclinical experiments indicated that *Ganoderma* *lucidum* possessed a broad spectrum of antibacterial and antiviral activities. Besides, in vitro or in animal models, polysaccharides or triterpenoids from *Ganoderma lucidum* exhibited activities against *Herpes simplex* virus, hepatitis B virus, HIV, and Epstein-Barr virus. Moreover, *Ganoderma* *lucidum* also contained antibacterial components inhibiting Gram-positive and/or Gram-negative bacteria. Serum HBV DNA and hepatitis B e antigen (HbeAg) levels of hepatitis B patients were significantly decreased after treatment with *G. lucidum* polysaccharides at 5400 mg/day for 12 weeks in a double-blind, randomized, placebo-controlled clinical study [80]. A 15 kDa antifungal protein, named ganodermin, was isolated from the medical mushroom *Ganoderma lucidum*. It was observed that ganodermin inhibited the mycelial growth of *Botrytis cinerea*, *Fusarium oxysporum* and *Physalospora* *piricola*, with an IC_50_ value of 15.2 µM, 12.4 µM and 18.1 µM, respectively [80].

In addition to the fruiting bodies, mushrooms mycelia could be used as functional foods and nutraceutical sources [81]. Two new benzoate derivatives and three new sesquiterpenoids were isolated from the mycelia of *Stereum hirsutum*. The benzoate derivatives showed antimicrobial activities against methicillin-resistant *Staphylococcus aureus*, with MIC values of 25.0 µg/mL. The research supported the notion that mycelia of *Stereum hirsutum* could be a functional food [82]. In addition, a study about the effect of dietary supplementation with white mushrooms on host resistance to influenza infections proved thus was not adequate to confer a protective effect against influenza infections [82]. The antimicrobial activities of some mushrooms not mentioned above are shown in Table 3.

## 7. Other Bioactivities of Mushrooms

In addition to the abovementioned bioactivities, mushrooms exhibit several other beneficial effects, such as hepatoprotective, antidiabetic, anti-hypercholesterolemia, and antihypertensive activities [89].

The protective effects of mushrooms on the liver were investigated widely. *Ganoderma* *lucidum* is an extensively used mushroom for the treatment of hepatopathy of various etiologies. In a study investigating the hepatoprotective activity of *Ganoderma* *lucidum* it was demonstrated that its extracts, which mainly contain polysaccharides or triterpenoids, protected the liver against injury caused by exposure to toxic chemicals (e.g., CCl_4_) or bacillus Calmette-Guerin plus lipopolysaccharide in preclinical studies. In addition, a randomized placebo-controlled clinical trial showed that hepatitis B e antigen (HbeAg) and HBV DNA levels in 25% patients were decreased significantly after treatment with *Ganoderma* *lucidum* polysaccharides for 12 weeks [79]. In another study, the hepatoprotective effects of *Ganoderma lucidum* aqueous extracts (GLE) were evaluated on liver injury induced by α-amanitin in mice. Compared with the α-amanitin control group, treatment with GLE significantly decreased serum ALT and AST levels, obviously increased SOD and CAT activities, and decreased MDA content in liver [90]. Besides, ganodermanondiol, a bioactive compound isolated from *Ganoderma lucidum*, was examined for the protective effects against *tert*-butyl hydroperoxide (*t*-BHP)-induced hepatotoxicity. The results demonstrated that ganodermanondiol exhibited potent cytoprotective effects on *t*-BHP-induced hepatotoxicity in human liver-derived HepG2 cells [91]. Except for *Ganoderma lucidum*, there are some other medicinal mushrooms possessing hepatoprotective effects. Polysaccharides from the mushroom *Russula vinosa* protect the liver from CCl_4_-induced hepatic damage via antioxidant mechanisms [92]. The mushroom *Antrodia camphorate* has anti-hepatic fibrosis activities in vitro [93]. Hepatoprotective activities, including anti-hepatitis, anti-hepatocarcinoma and anti-alcoholism, of mushroom *Antrodia cinnamomea* have been demonstrated both in vitro and in vivo [94]. In addition, consumption of mushroom *Phellinus linteus* could prevent tacrine-induced hepatotoxicity [95].

Type 2 diabetes mellitus, a disease with impaired glucose, protein and lipid metabolism, low-grade chronic inflammation, and immune dysfunction, is a global public health crisis [96]. Therefore, the anti-diabetic effects of mushrooms have been studied extensively. In one study, the effects of the mushroom *Pleurotus eryngii* on glycemic metabolism were examined in alloxan-induced hyperglycemic mice. Blood glucose and HbA1c were significantly decreased in the hyperglycemic mice (*p* < 0.05 and *p* < 0.01, respectively) after *Pleurotus eryngii* extract (100 and 200 mg/kg) oral administration to the mice over 5 weeks, while the level of insulin secretion was markedly elevated (*p* < 0.05). Besides, *Pleurotus* *eryngii* extracts treatment enhanced the body weight and significantly improved the concentration of hepatic glycogen in hyperglycemic mice (*p* < 0.05). The result suggested that mushroom *Pleurotus eryngii* possessed effects on glycemic metabolism by increasing glycogen and insulin concentrations as well as recovering injured β-cells and reducing free radical damage [97]. In another study, the antihyperglycemic effects of polysaccharides extracted from the medicinal mushroom *Inonotus obliquus* were also examined in alloxan-induced diabetic mice. Treatment with polysaccharide extracts (150 and 300 mg/kg body weight) resulted in a significant decrease in blood glucose levels, with percentage reductions of 16.64% and 20.09% at the 7th day, and 29.71% and 36.36% at the 21st day, respectively. Furthermore, serum contents of free fatty acids, total cholesterol, triglycerides, and low-density lipoprotein cholesterol were decreased significantly, whereas high-density lipoprotein cholesterol, insulin levels, and hepatic glycogen contents in the liver of diabetic mice were increased effectively. Concomitantly, a histological morphology examination revealed that the polysaccharides restored the damage of pancreatic tissues in mice with diabetes mellitus. These results showed that *Inonotus obliquus* exhibited antihyperglycemic, antilipidperoxidative, and antioxidant effects in alloxan-induced diabetic mice [98]. In another study, a polysaccharide-protein complex isolated from abalone mushroom *Pleurotus abalonus* showed antihyperglycaemic effects in alloxan-induced diabetic mice. This biomolecule protected the pancreas injured by oxidative stress, via elevating pancreatic insulin expression and lowering circulating glucose levels [99]. In addition, polysaccharides of oyster mushroom could be used as a dietary supplement for treatment of diabetics [100].

It was demonstrated that several medicinal mushrooms exhibited hypocholesterolemic effects. The effects of *Kluyveromyces marxianus* M3 isolated from Tibetan mushrooms on diet-induced hypercholesterolemia rats were investigated. Hyperlipidemic rats were treated with *K. marxianus* + HCD via oral gavage for 28 days. The results showed that levels of cholesterol, triglyceride, low-density lipoprotein cholesterol (LDL-C) in serum and liver and atherogenic index were meaningfully reduced (*p* < 0.01), and the high-density lipoprotein cholesterol (HDL-C) levels and anti-atherogenic index were significantly improved (*p* < 0.01) in rats. Furthermore, treatment of *K. marxianus* also decreased the build-up of lipid droplets in the liver and exhibited normal hepatocytes, which suggested a protective effect of Tibetan mushrooms in hyperlipidemic rats [101]. Besides, polysaccharides extracted from mushroom *Phellinus linteus* could significantly reduce the serum triglyceride, the blood cholesterol and serum LDL levels, and could increase HDL levels of the hyperlipemia mice. Thus, polysaccharides from *Phellinus linteus* have potential in the development of anti-hyperlipermia drugs [102].

Additionally, the water extract of the edible wild mushroom *Leucopaxillus tricolor* exhibited a clear antihypertensive effect on spontaneously hypertensive rats [103]. Besides, both *Cordyceps sinensis* and *Ganoderma lucidum* mushrooms show antinociceptive activity. Cordymin, a peptide purified from the mushroom *Cordyceps sinensis*, significantly inhibited the reaction time to thermal stimuli at 30, 60 and 90 min in the hot-plate test [33]. The agaricoglycerides extracted from mycelium of mushroom *Ganoderma lucidum* inhibited acetic acid-induced abdominal constrictions in mice in a dose-dependent manner (*p* < 0.5), inhibiting both phases of responses to the formalin test (*p* < 0.5), inhibiting the reaction time to thermal stimuli at 30, 60, and 90 min in the hot-plate test and showed strong activities against neurolysin (IC_50_ = 100 nM) in the neurolysin inhibition assay [104]. These results indicated agaricoglycerides extracted from *Ganoderma lucidum* mushroom might have potential applications as an analgesic medicine for human. Furthermore, it was observed that *Agaricus brasiliensis* presented anxiolytic effects on ischemia-induced anxiety rats [105]. In addition, there were several components showing inhibitory activities toward HIV-1 reverse transcriptase, including three laccases from *Pleurotus cornucopiae*, *Lentinus edodes* and *Ganoderma lucidum* [106,107,108], four lectins from *Hericium erinaceum*, *Russula delica*, *Pleurotus citrinopileatus* and *Stropharia rugosoannulata* [109,110,111,112], respectively.

## 8. Conclusions

Various mushrooms have been proved to possess numerous bioactivities, such as antioxidant, anti-inflammatory, immunomodulatory, anticancer, anti-diabetic, hepatoprotective, and antimicrobial activities. Therefore, numerous mushroom species have the potential to be developed into functional foods for the prevention and treatment of several chronic diseases, such as cancer, diabetes mellitus, hyperlipidemia, and hypertension. So far, popular mushrooms were evaluated extensively, but uncommon mushrooms were studied less frequently. Thus, the role of a large variety of mushrooms, especially unknown types, in human health is still a largely unexplored area of research. In the future, the bioactivities of some underutilized mushrooms should be evaluated comprehensively, and more bioactive compounds should be isolated and identified. A special attention should be paid to the mechanisms of action of the active components. Furthermore, some mushrooms possessing various health benefits should be developed into functional foods or medicines for prevention and treatment of several chronic diseases.

## Figures and Tables

**Table 1:** Antioxidant activities of some mushrooms.

Mushrooms	Bioactive Compounds	Antioxidant Activity	References
*Agaricus brasiliensis*	Crude Se polysaccharide and total soluble Se protein	Scavenging of DPPH and hydroxyl radicals	[24]
*Cortinarius purpurascens*	Rufoolivacin, rufoolivacin C, rufoolivacin D and leucorufoolivacin	Scavenging of DPPH radicals	[25]
*Ramaria flava*	Phenolic compounds	Scavenging of DPPH and OH radicals	[26]
*Phellinus baumii Pilat*	Polysaccharide	Scavenging of hydroxyl, superoxide and DPPH radicals	[27]
*Pleurotus abalonus*	Polysaccharide-peptide complex LB-1b	Exhibition of antioxidant activity in erythrocyte haemolysis	[28]
*Cordyceps taii*	Polysaccharides	Scavenging of DPPH, hydroxyl, and superoxide anion radicals, and enhancement of antioxidant enzyme activities	[29]
*Agaricus bisporus*	Polysaccharides, phenolics	Scavenging of superoxide, hydroxyl and DPPH radicals and hydrogen peroxide, enhancement of the activities of antioxidant enzymes in sera, liver, and heart of mice	[30,31]

**Table 2:** Anticancer activities of some mushrooms.

Mushrooms	Bioactive Compound(s)	Effects	References
*Taiwanofungus camphoratus*	5-(Hydroxymethyl) furan-2-carbaldehyde, 3-isobutyl-1-methoxy-4-(4′-(3-methylbut-2-enyloxy) phenyl)-1*H*-pyrrole-2, 5-dione	Inhibition of the proliferation of K562 and HepG2 tumor cells	[68]
*Tricholoma terreum*	Four meroterpenoids, terreumols A–D	Cytotoxicities against five human cancer cell lines	[69]
*Agrocybe aegerita*	Lectin	Inhibition of human and mouse tumor cells via inducing apoptosis	[70]
*Albatrellus confluens*	Grifolin	Inhibition of the growth of tumor cells by inducing of apoptosis and causing cell-cycle arrest	[71,72]
*Pleurotus eryngii*	Polysaccharides	Inhibition of tumor growth and increased relative thymus and spleen indices	[73]
*Tuber indicum*	Ribonuclease	Inhibition of the proliferation of hepatoma (HepG2) and human breast cancer cell lines (MCF7)	[74]
*Pleurotus abalonus*	Polysaccharide-peptide complex LB-1b	Inhibition of the proliferation of hepatoma HepG2 cells and breast cancer MCF7 cells	[28]

**Table 3:** Antimicrobial activities of some mushrooms.

Mushrooms	Microorganisms	References
*Agaricus bisporus*	*Neurospora sitophila*, phytopathogenic fungi	[83]
*Lenzites betulina*	*Staphylococcus aureus*, *Escherichia coli*, *Bacillus subtilis*, *Fusarium graminearum*, *Gibberella zeae* and *Cercosporella albo-maculans*	[84]
*Agrocybe cylindracea*	Several fungal species	[85]
*Tricholoma giganteum*	*Fusarium oxysporum*, *Mycosphaerella arachidicola* and *Physalospora piricola*	[86]
*Hericium erinaceus*	*Helicobacter pylori*	[87]
*Clitocybe sinopica*	*Agrobacterium rhizogenes*, *A. tumefaciens*, *A. vitis*, *Xanthomonas oryzae* and *X. malvacearum*	[78]
*Pleurotus ostreatus*	*Fusaerium oxysporum*, *Mycosphaerella arachidicola* and *Physalospora piricola*	[88]
